# The Kynurenine Pathway: Unraveling Its Role in Neurological Disorders via Mammalian Cellular Models

**DOI:** 10.3390/ijms27146337

**Published:** 2026-07-16

**Authors:** Elizaveta S. Podshivalova, Sergey I. Kutsev, Aleksandr V. Shestopalov

**Affiliations:** 1 Research Centre for Medical Genetics, 115522 Moscow, Russia; sverepeeva@gmail.com (E.S.P.);; 2 Department of Biochemistry and Molecular Biology, Pirogov Russian National Research Medical University, 117513 Moscow, Russia

**Keywords:** tryptophan metabolism, kynurenine pathway, neuroinflammation, neurodegeneration, neurotoxicity, cellular models

## Abstract

The kynurenine pathway (KP) constitutes the primary route of tryptophan catabolism, generating a spectrum of neuroactive metabolites that profoundly influence central nervous system function. Dysregulation of the KP is increasingly recognized as a critical pathogenic mechanism underlying diverse neuropathological conditions. This review critically evaluates the most widely cited mammalian cellular models currently utilized to delineate the causal role of KP alterations in neurological disease. Specifically, this article examines primary cell cultures, immortalized and tumor-derived cell lines, stem cell-derived systems, and ex vivo organotypic brain slices and tissues, highlighting their distinct methodological advantages, translational limitations, and specific enzymatic profiles. Across the described cellular systems, a recurring mechanistic theme emerges: quinolinic acid-driven mitochondrial dysfunction, oxidative stress, and NAD+ depletion converge in neurodegenerative conditions such as Alzheimer’s disease, Huntington’s disease, and amyotrophic lateral sclerosis. Conversely, kynurenic acid exhibits disorder-dependent—and at times opposing—roles, attenuating dopaminergic neurotoxicity in Parkinson’s disease models while contributing to synaptic pruning deficits in schizophrenia models. Furthermore, cellular models demonstrate that IDO1/TDO induction and downstream metabolite shifts are frequently cell type- and species-dependent, complicating direct extrapolation to human pathology. Because no single experimental system achieves complete physiological fidelity, elucidating the complex dynamics of the KP and identifying novel therapeutic targets requires the integration of data across complementary platforms.

## 1. Introduction

The kynurenine pathway (KP) of tryptophan metabolism has emerged as a mechanistic nexus linking immune activation, neuromodulation, and cellular energy metabolism, positioning it as a tractable therapeutic target in neurological and neuropsychiatric disorders [1,2]. The KP produces a variety of biologically active metabolites that interact bidirectionally with multiple physiological systems, including the endocrine, hematopoietic, immune, cardiovascular, and nervous systems, while the final product of the KP, NAD^+^, is necessary for vital cellular processes [3].

**Figure 1:**
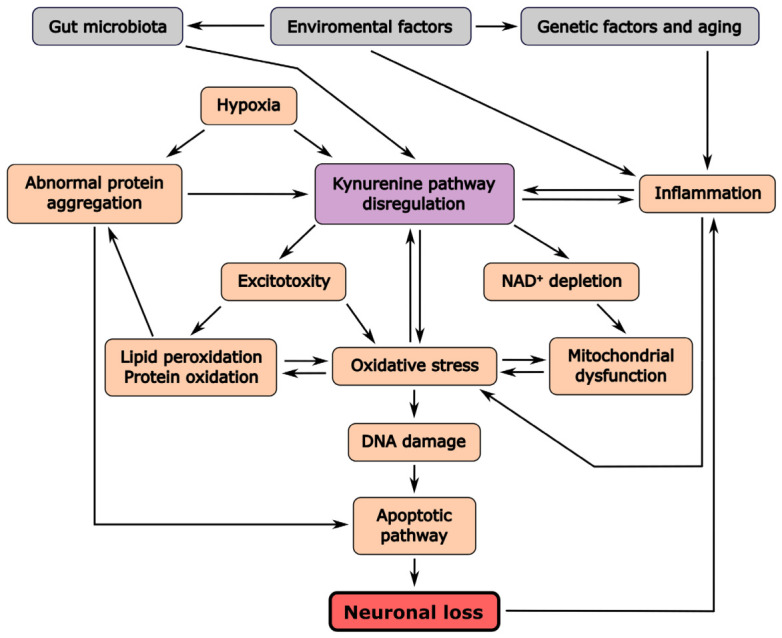
A schematic representation of the KP’s role in the cellular processes involved in neurodegeneration. Genetic, environmental, and microbiota-related factors converge on neuroinflammation and KP dysregulation, which promote neuronal damage through excitotoxicity, NAD^+^ depletion, oxidative stress, and mitochondrial dysfunction, culminating in apoptosis and neuronal loss. Gray indicates factors that contribute to the development of the pathological processes shown in orange. Minor and secondary interactions are simplified for clarity. NAD^+^, nicotinamide adenine dinucleotide.

Neurodegenerative and neuropsychiatric disorders constitute one of the most significant challenges in contemporary medicine, affecting hundreds of millions of people worldwide and imposing a substantial socio-economic burden on society. Diseases such as Alzheimer’s disease (AD), Parkinson’s disease (PD), Huntington’s disease (HD), amyotrophic lateral sclerosis, multiple sclerosis, and neuropsychiatric conditions including schizophrenia, major depressive disorder (MDD), and bipolar disorder share a common yet incompletely understood feature: profound dysregulation of neuroimmune homeostasis and altered neurotransmitter metabolism [4,5,6]. Despite the various clinical manifestations that range from dysfunction to neuronal death, these conditions are united by evidence of neuroimmune dysregulation, though the nature, magnitude, and causal role of neuroinflammatory processes differ substantially across diagnostic categories [7,8]. In recent decades, accumulating evidence suggests that dysregulation and imbalances in the metabolites of the KP of tryptophan catabolism constitute a critical mechanistic link between neuroinflammation and neurodegeneration [9]. Factors such as aging, genetics, and environmental influences initiate the processes accompanying the development of neurological disorders and modulate KP enzyme expression and metabolite flux in a hypoxia-inducible factor 1-alpha (HIF-1α)-dependent manner [10], through proinflammatory cytokine signaling, particularly via IFN-γ [11], and through the upregulation of key enzymes by amyloid-β (Aβ) and hyperphosphorylated tau [12,13]. Additionally, gut microbiota dysbiosis represents a further upstream modulator of KP activity [14]. Altered KP metabolism contributes to the development of mitochondrial dysfunction and oxidative stress, which, together with hypoxia, inflammation, and abnormal protein aggregation, drive neuronal loss (Figure 1) [15].

Despite the compelling and consistent associations observed in clinical studies—including changes in QUIN and KYNA levels in cerebrospinal fluid of patients with AD, HD, depression [16,17,18,19], increased IDO1 expression in hippocampal sections in AD [20], and altered KYN/TRP ratios in schizophrenia [21]—correlational data from human cohorts are contradictory and have limited capacity to establish causality, dissect mechanisms, or evaluate therapeutic interventions. Human studies are confounded by disease heterogeneity, comorbidities, medication effects, and the inaccessibility of the living brain to direct biochemical interrogation. It is precisely for these reasons that well-established experimental models, including cell-based models—ranging from primary cultures and organotypic preparations to in vitro cell culture systems—are indispensable tools for advancing our mechanistic understanding of the KP’s involvement in neurological disease. Such models enable precise manipulation of individual enzymatic steps and targeted pharmacological and genetic modulation at defined nodes of the pathway, as well as directly evaluating the subsequent phenotypic, biochemical and functional consequences, thereby enabling causal inference that cannot be drawn from correlational human data alone.

The present review appraises the cellular experimental models of mammalian origin most frequently employed to elucidate the role of the KP in major neuroinflammatory, neurodegenerative, and neuropsychiatric disorders—primary cultures, immortalized and tumor-derived cell lines, stem cell-derived systems, organotypic brain slice cultures (OBSCs) and ex vivo preparations—with emphasis on the model-specific features, strengths, and limitations of KP expression and metabolism in each system, an essential prerequisite for correctly interpreting what these models reveal about the pathophysiological role of the KP in neurological disease and for guiding the development of KP-targeted therapeutic strategies (Table 1).

## 2. Literature Search Strategy

For this review, a comprehensive literature search was conducted in the PubMed, Scopus and Google Scholar databases up to 25 June 2026 using various combinations of keywords. Representative search queries included the terms “kynurenine”, “kynurenine pathway”, as well as the names of individual KP metabolites and enzymes, combined with keywords related to specific neurological disorders (e.g., “Alzheimer’s disease”, “Parkinson’s disease”, “schizophrenia”, and “major depressive disorder”), cellular systems (e.g., “primary culture”, “microglia”, “astrocytes”, “neurons”, “glia”, “organoids”, “iPSC”, and “SH-SY5Y”), and mechanistic processes (e.g., “neuroinflammation”, “oxidative stress”, “AhR”, “mitochondrial dysfunction”, and “immune regulation”). No publication date restrictions were applied in order to ensure full coverage of the relevant literature. Studies not directly focused on both neurobiological disorders and various aspects of the kynurenine pathway were excluded. The reference lists of relevant articles were also screened to identify additional pertinent studies. In total, 251 sources were selected for inclusion in the review.

## 3. The General Characteristics of the Kynurenine Pathway

**Figure 2:**
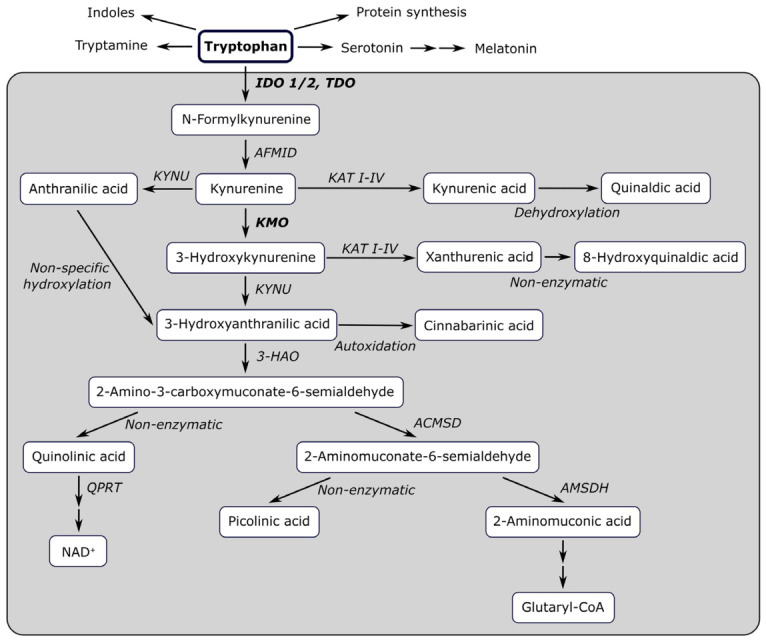
Tryptophan degradation via the kynurenine pathway in mammalian cells. The key enzymes of the KP, which are the most extensively studied and frequently cited in the literature, are highlighted in bold. Abbreviations: IDO 1/2, Indoleamine-2,3-dioxygenase 1/2; TDO, Tryptophan-2,3-dioxygenase; AFMID, Arylformamidase (also known as formamidase); KMO, Kynurenine 3-monooxygenase; KYNU, Kynureninase; KAT I-IV, Kynurenine aminotransferases I-IV; 3-HAO, 3-Hydroxyanthranilate-3,4-dioxygenase; ACMSD, Aminocarboxymuconate semialdehyde decarboxylase; AMSDH, Aminomuconate semialdehyde dehydrogenase; QPRT, Quinolinate phosphoribosyltransferase; NAD^+^, Nicotinamide adenine dinucleotide; CoA, coenzyme A.

The KP represents the predominant catabolic route for tryptophan, accounting for more than 95% of its systemic degradation (Figure 2) [22,23].

The initiating and rate-limiting reaction of the KP is the oxidative cleavage of the indole ring of L-tryptophan (TRP), catalyzed by three enzymes: indoleamine-2,3-dioxygenase 1 and 2 (IDO1, IDO2) and tryptophan-2,3-dioxygenase (TDO, also known as TDO2) [24]. IDO1 is the most broadly expressed of these enzymes, with a wide distribution across both immune and non-immune cells, including macrophages, microglia, dendritic cells, astrocytes, fibroblasts, and epithelial cells. IDO1 is regarded as the principal driver of kynurenine production under inflammatory conditions. IDO1 exhibits relatively broad substrate promiscuity, accepting not only L-tryptophan but also D-tryptophan, tryptamine, 5-hydroxytryptophan, and serotonin as substrates [25]. Beyond its enzymatic role, IDO1 functions as a signaling molecule that shapes immune cell phenotypes toward immunoregulatory states [26,27]. A defining characteristic of IDO1 is its inducibility: expression is potently upregulated by IFN-γ and other proinflammatory mediators, including TNF-α, IL-6, and IL-10 [28,29]. IDO2, by contrast, is comparatively insensitive to cytokine stimulation, displays a restricted expression pattern predominantly confined to the liver, kidney, and antigen-presenting cells (dendritic cells and B cells), and exhibits markedly lower catalytic activity toward TRP than IDO1 [30,31]. TDO, in turn, is constitutively expressed primarily in the liver and brain, where it plays a central role in maintaining systemic TRP homeostasis [32,33]. All these enzymes convert TRP to N-formylkynurenine, which is rapidly hydrolyzed to L-kynurenine (KYN)—the central branch point metabolite of the pathway. From KYN, the pathway diverges into several distinct branches of critical neurobiological importance. Kynurenine aminotransferases (KATs) I–IV convert KYN to kynurenic acid (KYNA), an endogenous antagonist of ionotropic glutamate receptors (NMDA and AMPA) and the α7-nicotinic acetylcholine receptor (α7-nAChR), with broad neuroprotective and anti-inflammatory properties [34,35], although the affinity for α7-nAChR has been questioned [36]. KYNA also acts as a ligand for the G-protein receptor 35 (GPR35) and the aryl hydrocarbon receptor (AhR), which are two signaling receptors that function in both the brain and peripheral organs [37,38]. AhR regulates the function of various cells in both the innate and adaptive immune systems, including processes related to inflammation and carcinogenesis [39,40]. On the other hand, the enzyme kynurenine-3-monooxygenase (KMO) initiates a cascade of reactions leading to the formation of neurotoxic metabolites such as 3-hydroxykynurenine (3-HK) and its metabolite, xanthurenic acid (XANA), and 3-hydroxyanthranilic acid (3-HAA) [41,42]. Although the biological effects of the less characterized metabolite XANA remain insufficiently characterized, it has been demonstrated to function as an endogenous modulator of glutamatergic neurotransmission, leading to a net reduction in extracellular glutamate levels [43]. Subsequently, the KP again diverges into several metabolic branches. The unstable intermediate 2-amino-3-carboxymuconate-6-semialdehyde undergoes either enzymatic decarboxylation by ACMSD or spontaneous non-enzymatic cyclization to form quinolinic acid (QUIN), which is an endogenous agonist for NMDA receptors. This compound has a pronounced neurotoxic effect through overactivation of glutamatergic signaling and calcium-dependent toxicity [44,45]. In the human brain, ACMSD isoform I is expressed at low yet detectable levels relative to kidney and liver, whereas ACMSD isoform II is absent [46,47]. The terminal product of the pathway, nicotinamide adenine dinucleotide (NAD^+^), is an essential cofactor in cellular energy metabolism, DNA repair, and sirtuin-mediated signaling, thereby linking KP activity to mitochondrial function and cellular survival [48]. An alternative route, the glutarate pathway, leads to the formation of glutaryl-CoA, acetyl-CoA, and glutaric acid [49]. Picolinic acid (PIC) is formed via non-enzymatic cyclization in this pathway from an unstable intermediate metabolite and may have neuroprotective effects through inhibition of excitotoxic damage [50]. However, the exact mechanism of this process has not been fully elucidated [45,51]. Another less studied metabolite of the KP is cinnabaric acid (CA), formed as a result of oxidative non-enzymatic dimerization of 3-hydroxyanthranilic acid and found in trace amounts in the human brain [52]. Data indicate that CA possesses neuroprotective properties and is capable of modulating neurotransmission [53,54]. CA is an AhR ligand that stimulates the differentiation of human and mouse T cells producing IL-22 [55]. In addition to the aforementioned branches, there is an alternative pathway from KYN to 3-HAA, bypassing the enzyme KMO through anthranilic acid (AA). This alternative pathway competes with the KMO-dependent pathway and can alter the metabolic balance, affecting the production of a neurotoxic metabolite, 3-HAA [56,57]. Quinaldic acid (QUINA) and 8-hydroxyquinaldic acid (8-OH-QUINA) are poorly characterized metabolites of the KP, derived from KYNA and XANA, respectively [58,59]. Although administration of QUINA and 8-OH-QUINA does not affect the electrocorticogram in rats, these compounds may exhibit partial functional similarity to KYNA in physiological and pathological contexts [60,61,62].

The balance between all these branches and metabolites, and particularly the KYNA/QUIN ratio, has been proposed as a key determinant of neuronal vulnerability or resilience in the context of disease. Most of the metabolites produced from KYN have poor permeability through the blood–brain barrier, implying that the concentrations of these metabolites in the central nervous system (CNS) are predominantly regulated by local enzyme activity [63]. However, KYN is actively transported into the brain through the large neutral amino acid transporter (L-amino acid transporter 1/LAT1) [64]. Due to the relatively low basal activity of the enzymes TDO, IDO1, and IDO2 in the brain, approximately 60% of KYN found in the brain typically originates from the bloodstream. However, the local biosynthesis of this compound substantially increases when the activity of brain cells’ IDO is stimulated by an activated immune system [56].

A conceptually important feature of KP biology in the CNS is the pronounced cell-type specificity of enzyme expression, which has fundamental consequences for the spatial distribution of neuroprotective versus neurotoxic metabolites. Microglia predominantly express KMO and 3-HAO, rendering them the primary source of QUIN and 3-HK upon inflammatory activation [65,66]. Astrocytes, by contrast, highly express KATs—particularly KAT II (also known as AADAT), the dominant KAT isoform in the brain—and thus serve as the principal producers of neuroprotective KYNA [67]. Astrocytes also exhibit high expression of nicotinic acid phosphoribosyltransferase while maintaining negligible QPRT expression. Consequently, astrocytes preferentially engage the nicotinic acid rather than the quinolinate branch of the pathway to buffer against the functional consequences of oxidative stress under conditions of reduced NAD^+^ availability—an arrangement that further underscores their neuroprotective role within the CNS [68,69]. Neurons themselves have limited capacity for KP metabolism and are accordingly net consumers, rather than producers, of these metabolites [70]. This cellular compartmentalization implies that the local neuroimmune context—specifically the relative activation state of microglia versus astrocytes—determines the net neurochemical outcome of KP engagement [71]. Disruption of this cellular equilibrium, as occurs in chronic neuroinflammation or acute neuropsychiatric episodes, can shift the microenvironmental balance profoundly toward excess QUIN and deficient KYNA, creating conditions permissive for excitotoxic damage, synaptic dysfunction, and progressive neuronal loss [72]. Notably, KYN, at concentrations similar to those produced by astrocytes, induces a substantial production of QUIN in macrophages. These results suggest that astrocytes themselves have neuroprotective properties, reducing QUIN formation and increasing the synthesis of KYNA. Nevertheless, it is possible that during neuroinflammatory conditions, in the presence of macrophages and/or microglia, astrocytes may become indirectly neurotoxic due to the production of high KYN levels, which can be metabolized into the neurotoxic QUIN by nearby or infiltrating monocytic cells [73].

In addition to QUIN, other metabolites of KP have pro-neuroinflammatory and neurotoxic effects. Even relatively low levels of 3-HK and 3-HAA can induce neurotoxicity through the generation of oxidative stress [74,75,76,77]. At millimolar concentrations, PIC acts as a cofactor for macrophages, inducing the expression of macrophage inflammatory proteins 1-α and 1-β [78]. 3-HK significantly inhibits respiratory chain complexes I, II, and IV; 3-HAA inhibits complexes I and II; and AA inhibits complexes I–III—collectively inducing mitochondrial dysfunction and exerting cytotoxic effects. Simultaneously, the activity of the Na^+^, K^+^-ATPase remains unaffected in the presence of these metabolites [79].

However, the biological effects of KP metabolites are highly context- and concentration-dependent and resist simple classification as exclusively neuroprotective or neurotoxic. QUIN, widely regarded as an excitotoxic NMDA receptor agonist, nonetheless participates at physiological concentrations in cellular metabolism, NAD^+^ biosynthesis, and neurodevelopmental processes such as neuronal growth and synaptogenesis; pathological accumulation, by contrast, drives oxidative stress, mitochondrial dysfunction, and excitotoxic injury [80]. KYNA presents an analogous duality: despite its established role as a neuroprotective antagonist of glutamatergic signaling, excessive concentrations impair synaptic plasticity, neurotransmission, and cognitive function [81,82]. Likewise, metabolites such as 3-HK and 3-HAA exhibit dual redox properties, capable of exerting either antioxidant or pro-oxidant effects depending on the cellular microenvironment and inflammatory state [83]. The net functional impact of the kynurenine pathway on the nervous system is therefore governed not by the absolute level of any single metabolite, but by their relative balance, regional distribution, cell type-specific production, and the temporal dynamics of immune activation.

## 4. Primary Cell Cultures

Primary cell cultures provide the closest available approximation to physiological KP activity for cell type-specific investigations, enabling direct measurement of enzyme expression and metabolite profiles under defined experimental conditions—though significant limitations must be considered. The primary cell culture models described in the literature can be broadly categorized into neuronal, astrocytic, microglial, and human or animal-derived cultures. Primary cell cultures are used to measure metabolite levels, the expression of enzymes involved in the KP, and to conduct targeted modulation experiments, for example using IFN-γ or lipopolysaccharide (LPS). The use of primary mammalian neurons is constrained by the fact that, following terminal differentiation into mature neurons, cells lose their proliferative capacity. Intraspecies biological variability increases the sample sizes required to achieve adequate statistical power, substantially reducing the scalability of primary cultures for high-throughput screening applications; interspecies differences further complicate the interpretation of results by limiting their extrapolation across experimental systems and their translational relevance to human pathology.

An additional limitation is the pronounced cell type-specific organization of the KP in the brain: the use of single-cell-type primary cultures does not capture intercellular interactions that modulate KP activity. This limitation can be partially mitigated by co-culture systems. Using IFN-γ-stimulated purified cultures of human neurons, astrocytes, and microglia, it has been indicated that all three cell types express IDO; however, only microglia produce detectable amounts of QUIN [84]. Using primary cultures of fetal human astrocytes, divergent effects of QUIN across a concentration range spanning low physiological to high excitotoxic levels have been demonstrated. QUIN induced IL-1β production, astrogliosis, and inhibition of glutamine synthetase activity. At low concentrations (50 nM), QUIN treatment elicited a concomitant increase in glial fibrillary acidic protein (GFAP) levels and reduction in vimentin expression—a pattern consistent with reactive astrogliosis. At pathophysiological concentrations (>500 nM), QUIN induced a dose-dependent reciprocal shift in structural protein expression, characterized by upregulation of vimentin accompanied by a concurrent reduction in GFAP expression [85]. In another study. the decrease in cellular NAD^+^ levels correlated inversely with increasing extracellular lactate dehydrogenase activity in a dose-dependent manner at QUIN concentrations greater than 150 nM in human fetal astrocytes and neurons over 24 h. Concomitantly, upregulation of both mRNA and protein expression of the inducible (iNOS) and neuronal (nNOS) isoforms of nitric oxide synthase was observed [86].

In addition to the three principal types of primary neural cells, studies of the KP in neuroinflammatory processes have also examined the contribution of human peripheral blood mononuclear cells and oligodendrocytes. During the development of an immune response, activated macrophages can infiltrate the CNS through a compromised or regulated blood–brain barrier, influencing cellular processes in the brain and activating microglia [87,88]. It has been demonstrated that, following IFN-γ stimulation, macrophages—but not lymphocytes—produce de novo approximately 20-fold higher levels of QUIN than microglia [65,89]. IDO1-expressing monocytes activate the IDO1–KYN–AhR axis, exerting disease-specific immunomodulatory effects in PD [90]. In contrast, oligodendrocytes appear to exert a neuroprotective role by producing KYNA, likely due to the absence of IDO and TDO expression under basal conditions and after IFN-γ treatment [91]. The absence of these two regulatory KP enzymes in oligodendrocytes may be associated with heightened susceptibility to alloreactive T cells, given that IDO1 plays a pivotal role in immune regulation—most notably in the suppression of T-cell proliferation [92]. Nevertheless, the biological function and activity of the KP in this cell type remains poorly characterized. Notably, subdural infusion of KYNA in rats induces demyelination without oligodendrocyte necrosis, accompanied by pronounced local astrogliosis and in the absence of a cellular inflammatory response [93].

In contemporary studies of the KP in neuropsychiatric and neurodegenerative disorders, primary rat cultures are used considerably less frequently, despite the fact that a number of foundational studies in this field were performed in rat models. This decline likely reflects several factors. First, the mouse represents a more tractable model for genetic manipulation, enabling detailed investigation of the regulation of key KP enzymes such as IDO and KMO. Second, most modern experimental protocols, including primary culture techniques and high-throughput “omics” approaches, have been standardized for mouse models. Finally, the widespread use of mice in neuroimmunology further contributes to their predominance in KP research, given the close link between this metabolic cascade and immune regulation. At the same time, species-specific differences in metabolism and cellular responses in rats should be taken into account, as they may limit the extrapolation of findings to humans. For example, in rats, systemic inflammatory stimulation with LPS and immune activation do not result in a significant increase in QUIN levels in the brain, most likely due to the lack of upregulation of IDO and TDO expression [94,95,96]. Nevertheless, primary rat cultures continue to appear in KP-focused studies published after 2015. For instance, KYNA has been demonstrated to enhance dendritic arborization complexity and promote synaptogenesis in primary mature rat cortical neurons, as well as to protect against the reduction in neuronal structural complexity and the loss of co-localized synaptic marker expression induced by conditioned medium from IFN-γ-stimulated mixed glia and by direct QUIN treatment of neuronal cultures [97]. In another study investigating the relationship between disrupted copper metabolism and neurodegenerative conditions—including AD, PD, Menkes disease, and Wilson’s disease—co-incubation of CuSO_4_ with 3-HK or 3-HAA was shown to potentiate copper-induced mitochondrial membrane potential reduction, decrease in glutathione levels and viability of primary rat cortical astrocytes, while concurrently abrogating copper-induced ROS generation [98]. In contrast, the laboratory mouse has become the predominant model organism in contemporary KP research owing to its tractability for genetic manipulation, the availability of standardized experimental protocols, and its widespread use in neuroimmunological studies. At the same time, important interspecies differences in KP regulation and immune responses remain relevant when extrapolating findings to humans [99]. Primary mouse cultures more closely resemble human KP regulation than rat cultures and are cited more frequently. As in humans, immune activation in mice induces IDO in the brain, followed by increased QUIN production by microglia [100,101]. Examination of primary mouse neuronal, astrocytic, and microglial cultures revealed KAT II expression across all three cell types [102].

Within primary culture models of the KP, microglia occupy a particularly important position. In contrast to peripheral immune cells such as bone marrow-derived macrophages and monocytes, primary human microglia remain insufficiently characterized. This is due to several factors: first, they differ substantially from microglia of model organisms, particularly the house mouse, in both transcriptomic and functional profiles; second, their isolation from human tissue is limited by availability and ethical constraints; and third, existing isolation and culture methods likely alter their key properties [103]. In monoculture, microglia typically adopt an unramified, amoeboid morphology resembling that observed in damaged tissue and rapidly dedifferentiate ex vivo, losing their characteristic gene expression profile [104].

Mouse microglia demonstrate partial similarity to human microglia, and protocols exist for isolating pure microglial cultures from both neonatal and adult mouse brains [105,106]. However, overall, mouse microglia are less reactive, both in terms of secretory responses—to LPS, IL-1β, IFN-γ, or TNF-α—and phagocytic capacity. In particular, mouse microglia exhibit robust induction of nitric oxide (NO) secretion in response to IFN-γ or LPS—a response not observed in primary human microglia—whereas iNOS inhibition was found to exert no effect on IDO expression [107,108,109]. The effects of NO secretion on the remaining KP enzymes have not been evaluated.

## 5. Immortalized and Tumor-Derived Cell Lines

A critical confound in the use of immortalized and tumor-derived cell lines for KP research is the origin-dependent distortion of enzyme expression profiles: the provenance of these lines imposes constitutive patterns of KP enzyme activity that deviate substantially from those of primary adult brain cells, and these deviations are line-specific.

The origin of a cell line is also relevant in the context of age-related differences in TRP metabolism and KP activity in particular [110,111]. Cells of embryonic origin exhibit differences in phenotype and functional responses compared to those derived from the adult human brain [112,113,114]. Consistent with this, studies in rats have demonstrated that the aging brain undergoes a coordinated shift in the KP activity, characterized by declining TRP levels and TDO activity alongside reduced QPRT activity, and a concomitant elevation in IDO activity and the accumulation of KYN, KYNA, QUIN, and PIC [115].

The advantages and limitations of the major immortalized and tumor-derived cell lines most frequently cited in the context of kynurenine pathway research are summarized in Table 2.

Among the cell lines used to model neuronal processes and widely employed in the study of the KP, the neuroblastoma line SK-N-SH and its subclone SH-SY5Y deserve particular attention. SK-N-SH was established in 1970 from a metastatic bone marrow biopsy from a 4-year-old female cancer patient. SH-SY5Y is a thrice-cloned subline of the neuroblastoma cell line SK-N-SH (SK-N-SH -> SH-SY -> SH-SY5 -> SH-SY5Y), deposited at the ATCC^®^ in 1970 by June L. Biedler [135]. SK-N-SH cells stimulated with IFN-γ have been shown to express IDO, TDO, KYNU, KAT-II and KMO, whereas IDO expression is absent in untreated SK-N-SH cells [70]. When SK-N-SH is employed as a model system, it is important to consider phenotypic heterogeneity and differential gene expression between cell subtypes within the line. Two principal cell subtypes can be distinguished within this neuroblastoma line: neuroblastic (N-type) cells and epithelial-like substrate-adherent (S-type) cells, with an intermediate (I-type) occasionally recognized as a third category [136]. N-type cells exhibit a morphology resembling neuroblasts and are positive for tyrosine hydroxylase (TH) and dopamine-β-hydroxylase, which are characteristic of catecholaminergic neurons, whereas epithelial-like S-type cells lack these properties [137].

The SH-SY5Y neuroblastoma cell line, which is enriched in N-type cells relative to the parental SK-N-SH culture, is the most widely cited in vitro tumor-derived model for studying neuronal processes, especially in the context of PD [118]. Using retinoic acid (RA)-based differentiation protocols, N-type SH-SY5Y cells can be directed toward a cholinergic neuronal phenotype, most likely of cortical identity, with a transcriptomic signature closely resembling that of neocortical brain tissue [138,139]. The use of additional differentiating agents produces distinct neuronal phenotypes and biochemical alterations [140]. For example, sequential treatment with retinoic acid followed by 12-O-tetradecanoylphorbol-13-acetate (TPA) promotes the acquisition of a dopaminergic neuronal phenotype and reduces cellular sensitivity to neurotoxic agents and neuroprotective substances [141,142]. Differentiated SH-SY5Y cells exhibit neurite outgrowth, generation of evoked action potentials, and functional currents mediated by NMDA receptors that are suppressed by MK-801 (an NMDA receptor antagonist), despite conflicting reports regarding the clear mRNA expression of NR1, NR2C, and NR2D subunits alongside weak expression of the NR2A and NR2B subunits [143,144]. NR2A and NR2B are the principal GluN2 subunits of the NMDA receptor and are associated with synaptic transmission as well as learning and memory functions [145].

SH-SY5Y cells are commonly employed as a neuronal model to investigate the effects of exogenous KP metabolite exposure on physiological and biochemical parameters, given that neurons are physiologically subject to paracrine influences from both glial cells and cells of the immune system. It has been demonstrated that low QUIN concentration (50–150 nm) induces neuritogenesis in SH-SY5Y neuroblastoma cells independently of NMDA receptor activation [146]. Conversely, supraphysiological QUIN concentrations (>4 mM) exert neurotoxic effects on SH-SY5Y cells, manifesting as elevated reactive oxygen species levels, reduced neurite density, and induction of apoptosis [147,148]. These opposing concentration-dependent effects may be attributable to excessive NR2B subunit activation by supraphysiological QUIN levels, which dysregulates downstream effectors—including CREB and BDNF—critically implicated in neuronal differentiation and neuritogenesis [149,150]. Furthermore, exposure of SH-SY5Y cells to near-physiological concentrations of 3-HK has been shown to produce no overt neurotoxic effect; however, mitochondrial fragmentation was observed alongside a significant increase in mitochondrial number. Supraphysiological levels of exogenous 3-HK application in vitro are associated with ROS-induced apoptosis [151].

A critical practical consideration is phenotypic drift: with increasing passage number, SH-SY5Y cultures tend to become enriched in S-type cells, which divide faster and outcompete N-type cells [116]. This represents a major source of reproducibility concerns in the literature: laboratories working with high-passage SH-SY5Y may be operating with a predominantly non-neuronal cell population. In addition, the activity of the glutarate branch of the TRP catabolism pathway in SK-N-SH and SH-SY5Y cell lines remains a subject of uncertainty. In the parental neuroblastoma cell line SK-N-SH, in contrast to primary neurons, the ACMSD enzyme is reportedly nearly absent, with no detectable production of picolinic acid, mechanistically shifting TRP metabolism toward the production of QUIN [70,152]. However, comparative metabolite profiling of the KP in SH-SY5Y cells reveals a tendency toward elevated picolinic acid levels upon differentiation with RA and BDNF [153]. Given that ACMSD is required for the formation of the picolinic acid precursor, 2-aminomuconate semialdehyde, these findings suggest the presence of basal ACMSD expression in SH-SY5Y cells. This discrepancy may stem from the fact that SH-SY5Y is an N-type-enriched subclone of SK-N-SH, or may reflect differences in the sensitivity of the analytical methods employed.

In the literature, various cell lines of glioblastoma and astrocytoma are used to model astrocytic processes. The A172 and U87-MG cell lines are of particular interest due to differences in the expression of key enzymes of the KP between them and primary astrocytes. The A172 cell line was established in 1972 from the brain tissue of a 53-year-old male patient diagnosed with glioblastoma [119]. A172 cells exhibit high expression and activity of KMO, as well as constitutive expression of IDO1, IDO2, and TDO, which makes this line the least representative of primary astrocytes, but nevertheless serves as a convenient model for studying the pharmacological modulation of enzymes involved in the KP [120,121,122]. The A172 cell line produces relatively high levels of KYN due to constitutively elevated TDO expression, which is dependent on the MEK/ERK pathway and remains unaltered following IFN-γ stimulation [154]. In accordance with observations in other glioma-derived cell lines, IFN-γ stimulation of A172 cells likely induces suppression of ACMSD expression, thereby diverting KP flux toward the neurotoxic QUIN branch—a pattern that deviates substantially from the metabolic profile of primary glial cells [155]. The extensive application of A172 cells in inhibitor screening studies has facilitated the development of complementary in silico modeling approaches [154,156].

The U87-MG cell line, also referred to as U87 cells, was obtained from a 44-year-old female patient at Uppsala University in 1966 and deposited at the ATCC in 1982. It was subsequently discovered, however, that the ATCC U87-MG line does not correspond to the original Uppsala University culture and represents an authentic human glioblastoma cell line of unknown origin [124,157]. Nevertheless, the ATCC U87-MG cell line remains the most widely used and versatile model, appearing in virtually all major studies in the field of neurological disorders. U87-MG cells display the lowest expression of KMO, constitutive TDO expression, and no detectable IDO1 or IDO2 basal expression [121]. Collectively, these features render U87-MG the closest approximation to primary astrocytes among the classical, widely employed glioblastoma cell lines. TDO-driven kynurenine accumulation promotes U87-MG cell proliferation and invasion via the AhR/AKT/CREB signaling axis and suppresses anti-tumor T-cell responses [158]. Hypoxia reversibly attenuates TDO production through an HIF-1α-dependent mechanism [156]. Treatment with IFN-γ induces IDO1 expression, promoting KYN release; both IDO1 induction and direct KYN treatment increase AhR expression and enhance its nuclear translocation [120,122,123]. Using the U87-MG model, activation of the KYN–AhR axis has been demonstrated to potentiate glioma invasion [122,158,159]. Furthermore, U87-MG cells have served as the experimental basis for demonstrating that KYNU is a functionally relevant enzyme whose inhibition suppresses complement activation, elicits a cellular stress response, and impairs glioblastoma cell viability [160].

In certain studies, A172 and U87-MG cultures are employed in parallel to interrogate shared features of glioblastoma KP biology. Using both lines, it was determined that activation of the AhR pathway leads to increased hpol κ mRNA and protein levels, thereby augmenting endogenous DNA damage in a manner likely contributing to the elevated replication stress and genomic instability characteristic of these tumors [161]. In a separate investigation, both A172 and U87-MG cells were found to express QPRT—in contrast to primary astrocytes—indicating that the quinolinate-dependent, rather than the nicotinate-dependent, NAD^+^ salvage pathway is preferentially operative in glioma cells. Additionally, the absence of 3-HAO expression in glioma cells, including A172 and U87-MG, implies that adequate intratumoral QUIN supply is contingent upon microglial paracrine provision in exchange for KYN secreted by tumor cells [69].

The immortalized cell line Human Microglia Clone 3 (HMC3) was established in 1995 via SV40-dependent immortalization of human embryonic microglia and was comprehensively characterized in a 2018 review [128]. Prior to authentication by ATCC, this culture had been used across different laboratories under alternative names, such as CHME3 and CHME-5; notably, there is evidence of cross-contamination of CHME-5 with rat glioblastoma cells [128,162]. This observation, together with the increasing use of HMC3 cells as a promising experimental model for elucidating the role of microglia in human disease, underscores the importance of employing authenticated cell lines and exercising caution when interpreting data obtained using this model. With respect to studies of the KP, HMC3 cells are frequently used to model pathological processes associated with AD [163,164,165,166]. In HMC3 cells, IFN-γ treatment markedly activates the KP, significantly reducing TRP levels while increasing concentrations of KYN, KYNA, AA, and 3-HAA; in contrast, LPS treatment does not induce KP activation [126]. It has also been shown that 3-HK promotes the induction of cellular senescence in HMC3 cells, stimulating the secretion of the neuroinflammatory cytokine IL-6 and increasing the expression of senescence markers p21 and p19, as well as senescence-associated β-galactosidase activity [166,167]. In another study, 3-HK significantly reduced the viability of HMC3 cells by inducing concentration-dependent pyroptosis via the mitochondrial pathway, leading to the release of the proinflammatory cytokine IL-1β [168]. However, transcriptomic analyses indicate that HMC3 cells more closely resemble astrocytic expression profiles than those of microglia [127]. Compared with primary microglial cells, HMC3 cells exhibit a phenotype resembling that of human pericytes and do not stain positively for any of the three commonly used microglial markers (Iba1, CD45, and PU.1) in vitro or in situ, instead expressing markers of vascular wall cells (PDGFRβ and NG2) [108]. These findings indicate that results obtained using the HMC3 model should be extrapolated to microglia with caution.

The murine microglial cell line BV2 represents another widely used in vitro model for studying neuroinflammation and the role of the KP in microglia. BV2 cells were generated in 1990 from primary microglia of neonatal C57BL/6 mice infected with a v-raf/v-myc retrovirus [169]. LPS stimulation of BV2 cells recapitulates, albeit to a lesser extent, responses observed in primary microglia, including increased expression of IDO1 and KMO and elevated levels of KYN [132]. 17β-estradiol attenuates LPS-induced upregulation of IDO1 expression in BV2 cells, an effect that is abrogated by previous treatment with small interfering RNA ERβ [170]. However, another study comparing gene expression profiles of primary microglia and BV2 cells demonstrated that the latter lack the inflammation-induced microglia-specific transcriptomic signature triggered by LPS, raising concerns about the translatability of such findings [133]. Experiments using BV2 microglial cells demonstrated that overexpression of miR-132 led to a reduction in QUIN levels without altering the activation status of microglia [130]. In BV2 cells, stimulation with Aβ has been demonstrated to increase QUIN levels relative to control cells, thereby activating microglia. Elevated QUIN concentrations impair mitophagy by inhibiting mitolysosome formation in microglial cells, leading to mitochondrial dysfunction and the accumulation of damaged mitochondria, which in turn contributes to cellular senescence and age-related neurodegenerative processes [134]. However, KYNA co-treatment reduced the proinflammatory cytokines TNF-α, IL-6 and Aβ phagocytosis [171]. IDO1 inhibition reduces BV2 cell size and filopodial extension, fluid uptake, and both macropinocytic and phagocytic activity, while concomitantly increasing IL-1β secretion and suppressing NLRP3 expression [172]. Exposure to O_3_ enhanced BV2 cell viability under QUIN treatment conditions without inducing oxidative or nitrosative stress or DNA damage, suggesting potential utility of ozone-based therapeutic approaches in neuroinflammatory disease [173]. IDO1 inhibitors (1-MT and berberine) markedly attenuated QUIN secretion in BV2 cultures and completely abrogated oligodendrocyte apoptosis in co-culture conditions [174]. Conditioned medium derived from IFN-γ-pretreated BV2 cells attenuated both neurite outgrowth and arborization complexity in primary cortical neurons isolated from rat pups [129].

Nevertheless, species-specific differences in KP expression between human and murine cell cultures may be greater than expected for an evolutionarily conserved pathway [94,95,175]. Illustrating this point, two murine conditionally immortalized oligodendrocyte cell lines differing in their degree of maturation not only exhibited distinct KP expression profiles from one another, but also diverged markedly from their human counterparts in key aspects of KP regulation. Notably, despite their mutual differences, both lines constitutively expressed TDO and upregulated its mRNA in response to IFN-γ—responses absent in human oligodendroglia [82,91,174]. It should be noted, however, that these interspecies discrepancies may be partly attributable to the conditionally immortalized status of the cell lines examined, rather than reflecting intrinsic species-specific differences in KP regulation per se.

## 6. Stem Cell-Derived Models

Induced pluripotent stem cell (iPSC)-derived and human embryonic stem cell (hESC)-derived cellular models, as well as organoids, exhibit substantially greater similarity to primary cultures and are therefore preferred due to their enhanced translational potential. iPSC-based models can be employed for the identification of candidate therapeutics using high-throughput screening approaches [176]. However, despite the increasing accessibility of stem cell–derived brain models, they remain relatively labor-intensive and costly compared with immortalized and tumor cell lines, and are still infrequently used in studies of the KP [114,177]. Among the key findings obtained using iPSC- and hESC-derived models in the context of KP research are those related to AD and schizophrenia.

It is important to consider KP activity in undifferentiated stem cells. Undifferentiated hESCs and iPSCs express key KP enzymes, including the rate-limiting enzymes IDO 1/2 and TDO, whose activity is associated with KYN production and release into the extracellular environment [114,178]. KYN acts as a paracrine signal that maintains stem cells in an undifferentiated state via activation of the AhR; the KYN–AhR complex further promotes the expression of IDO1 and AhR, establishing a positive feedback loop. Activation of KAT II and a decrease in KYN levels serve as biomarkers of differentiation [179]. Differentiation status, as well as patient-specific features, influence KP activity in iPSC-derived neurons (iNeurons), astrocytes (iAstrocytes), and microglia (iMicroglia). A comparative analysis of KP metabolite levels in human iAstrocytes derived from individuals with normal cognitive status and from a patient with late-stage AD revealed elevated KYN levels in patient-derived iAstrocytes. These cells also exhibited reduced glucose metabolism, which was restored to control levels following IDO1 inhibition [12]. Patient-specific iPSC-derived models of schizophrenia are currently being actively developed and utilized [180]. In iNeuron models derived from patients with schizophrenia carrying heterozygous deletions in *NRXN1*, increased expression of KAT III has been reported [181]. Although the use of growth factors to generate astrocytes, neurons, oligodendrocytes, and peripheral macrophages from stem cells began soon after the advent of iPSC technology, the generation of microglia proved more challenging [182]. iPSC-derived microglia (iMicroglia) have only been available since 2016 and their use has expanded rapidly, with numerous differentiation protocols now established [103,183,184,185]. Nevertheless, iMicroglia in monoculture tend to adopt an amoeboid morphology and exhibit spontaneous activation even in the absence of stimulation, requiring cues from neurons and astrocytes to maintain their identity and morphology [186].

In a human iPSC-derived dorsal forebrain organoid model with innately developing microglia, generated from patients with schizophrenia, induction of endogenous KYNA production and release led to a substantial decrease in microglial uptake of synaptic structures [187]. Using a murine cerebral organoid model, microplastic exposure has been demonstrated to elevate KYN and 3-HK levels, thereby promoting neurotoxicity through KP dysregulation [188].

The integration of organoid technology with CRISPR/Cas9 genome editing has further advanced the application of iPSCs in the study of neurodegenerative diseases [189]. When employing animal iPSC-derived models, potential interspecies differences must be taken into account. Illustrating this point, temporal transcriptomic responses to LPS have been shown to differ substantially between murine and human iPSC-based systems [190].

## 7. Organotypic Brain Slice Cultures and Ex Vivo Preparations

OBSCs and postmortem brain slices represent a physiologically relevant three-dimensional model of the brain. OBSCs preserve all major CNS cell populations and can be prepared from various brain regions, including those implicated in neurodegenerative processes [191,192]. This model constitutes a suitable experimental system for studying the KP, as it retains intercellular interactions necessary for maintaining the balance of its metabolites. These cultures preserve three-dimensional cellular architecture, intercellular connectivity, tissue-specific transport and diffusion processes, and neuronal functional activity for up to six weeks in vitro [193]. Limitations of this model include the absence of a functional vascular system and blood–brain barrier, axotomy, limited ability to assess the contributions of individual non-neuronal cell populations, and considerable methodological heterogeneity across studies [194,195,196].

Postmortem human OBSCs are the most representative, albeit limited, model in which the KP is primarily studied under conditions preserving native tissue architecture without prior experimental manipulation [197,198]. OBSCs constitute a particularly tractable model for the comparative investigation of cell type-specific and region-specific heterogeneity in KP enzyme activity. Additionally, OBSCs, in conjunction with data on the levels of metabolites in the kynurenine pathway across different brain regions under neuropathological conditions, allow for the design of experiments involving targeted pharmacological modulation [199,200]. Analyses of postmortem samples from patients with various neuropsychiatric and neurodegenerative disorders have revealed increased levels of 3-HK and QUIN in the neostriatum and cortex in Huntington’s disease, an elevated KYN/TRP ratio, and increased KYNA levels alongside altered KYNA/QUIN ratios associated with the severity of inflammatory responses in schizophrenia [21,201,202,203]. Inhibition of KMO attenuates post-ischemic neuronal death in organotypic hippocampal slice cultures [204]. Furthermore, in hippocampal sections from human AD brains, colocalization of TDO, QUIN, and Aβ-containing neurofibrillary tangles has been demonstrated [205]. However, the use of human organotypic slices is constrained by the need for ethical approval.

Animal brain tissue is more readily accessible; however, the region-specific activity of KP enzymes in such preparations remains incompletely characterized. In murine OBSCs, KAT II immunoreactivity was largely restricted to the soma, showing a perinuclear distribution in glial cells and partial extension into dendritic compartments in neurons [102,206]. Data from rat OBSCs indicate a predominant localization of KAT II in astrocytes, with little to no immunoreactivity detected in neurons or microglia [207]. This divergence in KAT II cellular distribution renders rat organotypic slices a less suitable model for investigating the KP in the context of human neurological disorders.

The greater availability of animal brain tissue enables experimental studies involving prior modulation of the KP. In organotypic slices of the rat dorsal raphe nucleus, administration of the proinflammatory factor IFN-γ reduced cell viability and increased IDO expression directly in neurons, rather than exclusively in microglia and astrocytes [208]. Moreover, animal slice models allow for the investigation of age-dependent dynamics of KP function, including differences between prenatal and postnatal periods, as well as the influence of maternal metabolites on fetal KP activity in utero. In mouse OBSCs, the prenatal period is characterized by predominance of the neuroprotective branch (KYNA), due to low KMO expression alongside a substantial contribution of KAT I, whereas a more balanced KP metabolism emerges postnatally [209]. In a separate study, ex vivo recordings of evoked local field potentials in coronal brain slices from mice born to dams fed a KYN-enriched diet during pregnancy revealed a prolonged contralateral response latency compared with the control group, indicating impaired white matter function [210]. In addition, studies frequently employ brain slices from genetically modified (knockout) animals, enabling validation of findings obtained in pharmacological experiments [205,209,211].

When employing OBSCs, consideration must also be given to potential alterations in KP activity arising during slice preparation itself—both as a consequence of postmortem biochemical changes and as a result of the capacity of CNS cells to mount adaptive structural and functional responses to mechanical injury and cellular stress, many of which are mediated through the KP [212,213].

## 8. Future Perspectives and Research Directions

Realizing the therapeutic potential of KP-targeted interventions will require reproducible, well-characterized experimental platforms whose complementary use can compensate for the limitations inherent to each model class.

The rapidly growing interest in iPSC- and hESC-derived models, coupled with their increasing accessibility and methodological maturation, is expected to substantially advance our understanding of the pivotal role of the KP in the pathogenesis of neurological disorders. A recently described iPSC-derived tri-culture system successfully recapitulated the transition from a homeostatic to a neuroinflammatory state, revealing dynamic interactions among microglia, astrocytes, and neurons during neuroinflammation [214,215]. This model represents a promising platform for dissecting the fine molecular mechanisms underlying KP regulation in neuropsychiatric and neurodegenerative conditions, surpassing the capabilities of earlier experimental approaches.

Among immortalized cellular models, the PC12 cell line serves as a potentially valuable yet underutilized system for KP research. PC12 cells were derived from a rat adrenal pheochromocytoma and established as a monoclonal line by Greene and Tischler in 1976 [216]. PC12 cultures exist in an undifferentiated state characterized by properties of chromaffin cells and the ability to synthesize catecholamines, primarily dopamine and norepinephrine. Upon treatment with nerve growth factor (NGF), these cells differentiate into neuron-like cells that are morphologically and functionally similar to sympathetic neurons and capable of synthesizing acetylcholine [217]. Differentiated PC12 cells extend neurites, express neuronal markers, exhibit electrical excitability, and are widely used to study neurodegeneration, apoptosis, neurotoxicity, and other neural processes [218,219]. The PC12 model has also been applied to AD research [220]. Evidence indicates that corticosterone-induced metabolic changes in PC12 cells resemble aspects of depression pathophysiology, with poorly differentiated PC12 cells representing a particularly suitable model for studying depressive states [221]. However, data on KP activity in PC12 cells remain scarce. High-performance liquid chromatography (HPLC) analysis of PC12 supernatants has confirmed active TRP catabolism and the presence of KP metabolites, including KYNA and QUIN [222]. IFN-γ stimulation increases IDO activity in PC12 cells [223]. Nevertheless, the rat origin of this cell line and interspecies differences in KP regulation between rodents and humans limit its translational applicability.

Despite the limited availability of human oligodendrocyte cultures, the paucity of data on KP activity in these cells may be partially addressed using rodent primary microglial models and, potentially, the oligodendrocyte cell line OLN-93 [224,225]. OLN-93 cells, derived from spontaneously transformed cells in primary rat brain glial cultures, retain expression of both KAT I and KAT II isoforms and are capable of synthesizing KYNA from exogenously supplied KYN [226,227].

Of particular note is a recent study in which a three-dimensional glioblastoma spheroid model was established incorporating all cell culture systems discussed in the present review: primary human astrocytes, and the U87-MG and HMC3 cell lines [228]. The resulting three-dimensional glioblastoma spheroids demonstrated structural stability and successfully recapitulated hallmark glioblastoma properties, including glial cell-dependent growth, invasion, and compactness, as well as the formation of a physical barrier restricting intercellular penetration. Moreover, the incorporation of astrocytes and microglia induced marked upregulation of IDO1, PTGES2, and PDL1 expression, indicative of glioblastoma stromal activation and the acquisition of immunosuppressive capacity—findings subsequently corroborated by the demonstrated suppression of NK-92 cell cytotoxicity against the spheroids. This model holds considerable promise for elucidating the reciprocal intercellular interactions mediated by KP activation in glioblastoma, and for high-throughput screening of KP-targeted inhibitors under conditions more faithfully recapitulating the complexity of the tumor microenvironment.

Beyond the model-specific methodological, ethical, biological, and translational limitations discussed in preceding sections, the interpretation of KP data across the cellular systems reviewed here is further constrained by general analytical sources of variability. With respect to gene expression analyses, whether or not total RNA is subjected to DNase treatment prior to reverse transcription can substantially affect results: omission of this step may yield spurious amplification products arising from genomic DNA when primers lack strict mRNA specificity, whereas its inclusion introduces additional sample processing steps that risk RNA degradation or material loss. The choice of qPCR primer pairs represents an additional source of discordance between studies, as primers differ in their sensitivity to alternative splicing and in the number of splice variants they amplify, in their dependence on RNA integrity as a function of amplicon length and proximity to the 3′ or 5′ end of the target transcript, and in amplification efficiency across commercial reverse transcription and qPCR kit combinations. HPLC-based quantification of KP metabolites is similarly affected by inter-laboratory variability, with published methods differing considerably in detection sensitivity and sample preparation procedures [229]. Furthermore, a substantial proportion of the available literature—including studies employing the cellular models reviewed here—has focused on a restricted set of KP nodes, most commonly the enzymes IDO, TDO, KMO, and KAT, and the metabolites KYN, KYNA, and QUIN, despite the considerably broader analytical capabilities afforded by contemporary analytical platforms. Collectively, variability in analytical methodology—spanning RNA sample preparation protocols and qPCR primer selection to the sensitivity of HPLC-based metabolite quantification and the breadth of metabolites measured—contributes substantially to inter-laboratory variability in KP data, compounding the model-specific limitations discussed above. Methodological standardization, encompassing harmonization of analytical protocols and a shift from targeted profiling of a restricted metabolite set toward comprehensive pathway assessment integrating metabolomic, transcriptomic, and functional readouts, is therefore a prerequisite for improving the reproducibility and translational potential of findings across the mammalian cellular model systems reviewed here.

Despite the growing interest in the KP and the availability of diverse experimental models, its role in the pathogenesis of neuropsychiatric and neurodegenerative disorders remains insufficiently understood, particularly in the context of rare diseases. Notably, there are currently no data on KP activity or metabolite levels in glutaric aciduria type I, a disorder caused by deficiency of glutaryl-CoA dehydrogenase (GCDH), despite the hypothesis that neurodegeneration in this condition may result from a metabolic shift toward QUIN production due to blockade of the glutarate pathway [230,231]. This gap is likely to be addressed in the near future with the development of relevant experimental models, including *GCDH* knockout cell lines and *GCDH*^−^/^−^ mouse models [232,233,234]. Existing reviews of lysosomal storage disorders, such as Krabbe disease, metachromatic leukodystrophy, Niemann–Pick disease type C, and Menkes disease, highlight the presence of neuroinflammation and/or neurodegeneration associated with these conditions. However, the role of the KP in their pathogenesis has not yet been investigated, despite their well-established genetic basis [235,236,237,238].

Future studies should also address the interplay between the kynurenine pathway and other metabolically integrated immunoregulatory systems, particularly polyamine metabolism. Both kynurenines and polyamines modulate neuroinflammatory responses, redox homeostasis, mitochondrial activity, and glial cell function, while simultaneously participating in AhR-mediated immune regulation [239]. Furthermore, polyamine-dependent processes such as spermidine-mediated eIF5A hypusination may represent an additional layer of translational and metabolic control relevant to cellular responses induced by KP activation [240]. Defining these interactions more precisely may improve our understanding of how metabolic stress programs contribute to neuronal vulnerability and neurodegeneration.

## 9. Conclusions

Cellular models of mammalian origin have been instrumental in establishing the KP as a causal—rather than merely correlative—contributor to neurological and neuropsychiatric disease. Taken together, the evidence reviewed here points to both convergent and disorder-specific mechanisms. QUIN-mediated excitotoxicity, mitochondrial dysfunction, and impaired NAD+ salvage recur as a shared mechanistic axis across Alzheimer’s disease, Huntington’s disease, and amyotrophic lateral sclerosis models, from primary neurons and astrocytes to postmortem tissue. In contrast, KYNA exhibits context-dependent duality: neuroprotective against dopaminergic cell death in Parkinson’s disease models, yet implicated in excessive microglial synaptic pruning in schizophrenia-derived iPSC and organoid systems—underscoring that a single metabolite cannot be assigned a uniform pathogenic or protective role across disorders. Glial activation (BV2, primary microglia, postmortem microglia) emerges as a common upstream driver of IDO1/TDO2 induction and downstream QUIN/KYN accumulation in AD, MDD, multiple sclerosis, and amyotrophic lateral sclerosis, whereas tumor-derived models (U87-MG, A172) reveal a distinct, QPRT-dependent NAD+ salvage strategy that supports glioblastoma invasion via the KYN–AhR axis rather than classical neurodegenerative excitotoxicity. Table 3 summarizes the alterations in the KP across various neuropathological conditions, as characterized in the mammalian cellular models reviewed herein.

No currently available model system fully combines physiological fidelity, mechanistic tractability, and scalability. Primary cultures most closely approximate native KP enzyme expression but are constrained by donor/tissue availability, limited proliferative capacity, and pronounced inter- and intraspecies variability; immortalized and tumor-derived lines offer reproducibility and genetic tractability but frequently diverge from primary cells in basal enzyme expression (e.g., constitutive TDO activity and altered KMO/IDO profiles in glioma lines) and carry uncertain provenance in some cases (e.g., U87-MG); stem cell-derived models afford patient-specific and developmentally relevant readouts but remain comparatively costly and underutilized; and organotypic slice cultures preserve native tissue architecture at the expense of throughput and, for human tissue, availability. Notably, mouse-derived primary and immortalized systems more faithfully reproduce the human-like, IFN-γ-driven induction of IDO and QUIN production than rat-derived counterparts, which fail to upregulate IDO and TDO upon immune challenge—a species-specific divergence that should inform model selection in future studies. Several systems with demonstrated but underexploited potential—including the PC12 and OLN-93 cell lines, and the recently described iPSC-derived tri-culture and glioblastoma spheroid platforms—merit further validation and wider adoption.

Beyond model-specific limitations, interpretation of KP involvement in neuropathology across all systems reviewed here remains constrained by reliance on a narrow panel of pathway nodes (IDO, TDO, KMO, KAT; KYN, KYNA, QUIN) and by considerable inter-laboratory variability, including differences in RNA processing, qPCR primer design, and HPLC-based metabolite quantification. Furthermore, despite the breadth of models now available, the KP remains entirely unexplored in several genetically well-defined CNS disorders, including glutaric aciduria type I and the lysosomal storage diseases discussed above—gaps that are likely attributable to disease rarity rather than to any inherent unsuitability of existing modeling approaches. Addressing these limitations will require the combined use of complementary model systems, the development of disease-specific platforms (e.g., GCDH-deficient cell lines), and a shift from targeted profiling toward standardized, integrated multi-omics characterization of the KP across the cellular models discussed in this review.

## Figures and Tables

**Table 1:** A comparative overview of the cellular models employed in the study of the kynurenine pathway.

Cellular Model Type	Example Models	Advantages	Limitations
Primary cell cultures	Neurons, astrocytes, microglia, macrophages, monocytes, oligodendrocytes	Physiologically relevant KP enzyme expression and metabolite profiles. Cell type-specific profiling of IDO, TDO, KMO, KAT, and KYNU activity. Human-derived cultures provide directly translatable data. Co-culture systems partially recapitulate intercellular KP regulation between various type cells.	Ethical concerns for human cultures. Limited proliferative capacity. Absence of intercellular interactions within the KP in monoculture systems. Phenotypic drift during prolonged cultivation. Limited scalability for high-throughput screening. Batch-to-batch variability and donor heterogeneity limit reproducibility.
Immortalized cell lines	Cell lines: SK-N-SH, SH-SY5Y, U87-MG, A172, BV2, HMC3	Unlimited proliferative capacity; low cost; easy maintenance. Highly reproducible experimental conditions across laboratories. Amenable to high-throughput pharmacological screening of KP inhibitors. Well-characterized genomes; susceptible to stable genetic manipulation (knockdown, overexpression, CRISPR/Cas9). Established protocols for KP enzyme modulation and metabolite quantification.	Low physiological relevance. Origin results in constitutive aberrant expression of KP enzymes and secretion of metabolites that diverge substantially from primary brain cells. Phenotypic and genetic drift, accumulation of mutations with increasing passage number. For animal lines, interspecies differences limit the translational applicability.
Stem cell-derived models	iNeurons, iAstrocytes, iMicroglia, organoids	Indefinite self-renewal capacity combined with pluripotency enables derivation of any CNS cell type of interest. Uniquely suited for patient-specific and translational KP research. Compatible with CRISPR/Cas9-mediated genetic manipulation.	Ethical concerns for human iPSC and hESC. Differentiation protocols are technically demanding and associated with substantial resource expenditure, limiting experimental throughput. Inter-line and inter-batch variability in differentiation efficiency poses a significant challenge for generating sample sizes sufficient to achieve adequate statistical power.
Organotypic slices	OBSCs, postmortem ex vivo preparations	Preserves three-dimensional tissue architecture, intercellular connectivity, and all major CNS cell populations. The compromise between in vitro and in vivo systems for investigating the intercellular effects of the KP.	Ethical concerns for human brain tissues. Absence of functional vasculature and blood–brain barrier. Axotomy disrupts long-range neural connectivity. Considerable methodological heterogeneity across studies and potential alterations in KP activity during tissue preparation.

**Table 2:** Key features of the kynurenine pathway in various immortalized and tumor-derived cell lines.

Cell Line	Cell Line Origin	Advantages	Limitations	References
SH-SY5Y	The thrice-cloned subline of the neuroblastoma cell line SK-N-SH from a 4-year-old female cancer patient	Differentiate into neuron-like cells exhibiting cholinergic, dopaminergic, or noradrenergic phenotypes. Expresses key KP enzymes (IDO1, TDO, KYNU, KAT-II, KMO) upon IFN-γ stimulation.	Mechanistic bias toward QUIN production due to probable absence of ACMSD, limiting study of the neuroprotective branch. Accumulation of epithelial-like cells upon prolonged passaging.	[70,116,117,118]
A172	The glioblastoma cell line from a 53-year-old male patient	Homogeneous, rapidly proliferating populations amenable to high-throughput KP inhibitor screening. Highest KMO expression and enzymatic activity among commonly used GBM cell lines. Constitutive TDO expression enables study of the TDO branch independently of IDO induction.	Constitutively elevated expression of TDO and KMO, together with basal IDO1 expression and lack of expression of 3-HAO, distinguish this cell line from primary astrocytes.	[69,119,120,121]
U87-MG	The cell line from a 44-year-old female patient, likely with glioblastoma	Low basal KMO expression and activity, resembling primary astrocytes in this respect. Constitutive TDO and IDO 1/2 expression; IFN-γ stimulation significantly potentiates KP enzyme expression with reduction in the KYNA/KYN neuroprotective ratio.	The provenance of this cell line remains unverified: likely not derived from the originally reported patient. Constitutive TDO activity and KYN-AhR signaling. Lack of basal expression of 3-HAO.	[69,120,122,123,124,125]
HMC3	The SV40-immortalized human microglial cell line	Human origin. IFN-γ treatment robustly activates KP: significantly reduces TRP and increases KYN, KYNA, AA, and 3-HAA levels.	Transcriptomic profile more closely resembles astrocytes than microglia. Exhibits pericyte-like phenotype with expression of PDGFRβ and NG2. LPS stimulation does not significantly activate KP, unlike primary microglia.	[108,126,127,128]
BV2	Immortalized with a v-raf/v-myc retrovirus from primary microglia of neonatal C57BL/6 mice	LPS stimulation upregulates IDO1 and KMO, elevating KYN and QUIN levels. IFN-γ stimulation increases IDO, KMO, and KYNU expression with accumulation of 3-HK in conditioned media, reducing neurite outgrowth.	Murine origin: interspecies differences limit translational applicability. LPS-triggered responses are poorly representative of primary microglial biology. Lacks the LPS-induced microglia-specific transcriptomic signature of primary microglia.	[129,130,131,132,133,134]

**Table 3:** Features of the KP identified in mammalian cellular models of neurological disorders.

Neurological Disorders	Cellular Model	Alterations of the Kynurenine Pathway	References
Alzheimer’s disease	BV2	Aβ-induced QUIN elevation, impaired mitophagy (mitolysosome blockade), mitochondrial dysfunction, and cellular senescence.	[134]
		KYNA-mediated attenuation of Aβ-induced proinflammatory cytokines (TNF-α, IL-6) and suppression of Aβ phagocytosis.	[171]
	iAstrocytes	Elevated KYN levels in patient-derived iAstrocytes versus cognitively healthy donors; reduced glucose metabolism, restorable via IDO1 inhibition.	[12]
	Primary human neurons	Dose-dependent QUIN-induced tau phosphorylation (Ser199/202, Thr231, Ser396/404) and colocalization with hyperphosphorylated tau.	[241]
	Primary macrophages + primary microglia	Aβ1–42-induced QUIN production by human macrophages and microglia.	[242]
	Primary astrocytes	QUIN-induced IL-1β expression; dose-dependent astrogliosis-like GFAP increase/vimentin decrease at low concentrations, reversed at high concentrations; reduced glutamine synthetase activity.	[85]
	OBSCs	Colocalization of TDO and QUIN with Aβ-containing neurofibrillary tangles.	[205]
	Postmortem human tissue	Overexpression of IDO and QUIN in hippocampal microglia, astrocytes, and neurons, particularly around senile plaques; QUIN colocalization with neurofibrillary tangles.	[84]
Parkinson’s disease	Primary monocytes	IDO1–KYN–AhR axis activation in IDO1-expressing monocytes, exerting disease-specific immunomodulatory effects.	[90]
	SH-SY5Y and SK-N-SH	KYNA-mediated attenuation of MPP+-induced dopaminergic cell death via Bax downregulation and prevention of mitochondrial membrane potential collapse and cytochrome c release.	[243]
Huntington’s disease	Postmortem human and mouse tissue	Elevated 3-HK and QUIN levels in the neostriatum and cerebral cortex during early-stage HD.	[201,244]
	Primary rat striatal neurons	QUIN-induced striatal neuronal death; CDNF-mediated protection via IRE1α/XBP1 endoplasmic reticulum stress signaling activation.	[245]
	Postmortem human tissue	Decreased KYNA concentrations within the cerebral cortex.	[246]
Major depressive disorder	BV2	TLR4-mediated LPS-induced upregulation of IDO1 and KMO expression and QUIN accumulation, fully blocked by the TLR4 inhibitor TAK-242.	[247]
	Postmortem human tissue	Higher density of QUIN-positive microglial cells in the subgenual and anterior midcingulate cortices in severe depression versus controls.	[248]
Schizophrenia	iNeurons	Upregulated KAT III expression in patient-derived models harboring heterozygous deletions in the *NRXN1* gene.	[181]
	iPSC-derived organoids with endogenously developed microglia	Enhanced endogenous KYNA production/secretion, reducing microglial engulfment of synaptic structures.	[187]
	Postmortem human tissue	Increased TDO2 activity (mRNA, protein, and metabolite levels; 1.9-fold increase in KYN) with elevated density of TDO2-positive white matter glial cells.	[249]
Glioblastoma	U87-MG, A172	QPRT expression, indicating preferential quinolinate-dependent NAD+ salvage; KYN–AhR axis activation driving tumor invasion.	[69,158]
	3D tri-culture spheroid model (primary human astrocytes + U87-MG + HMC3)	Astrocyte/microglia incorporation-induced IDO1, PTGES2, and PD-L1 upregulation, reflecting an immunosuppressive stromal phenotype that suppresses NK-92 cytotoxicity.	[228]
Amyotrophic lateral sclerosis	Postmortem human tissue	Pronounced microglial activation (HLA-DR+), upregulated IDO expression, and elevated QUIN levels in the neurons and microglia of the motor cortex and spinal cord.	[250]
Multiple sclerosis	BV2 + primary oligodendrocytes	IDO1 inhibition (1-MT, berberine)-mediated reduction in BV2 QUIN secretion and rescue of co-cultured oligodendrocytes from apoptosis.	[174]
Bipolar disorder	Postmortem human tissue	Elevated KYN levels (1.8-fold overall; 2.1-fold in the subgroup with psychotic features), concurrent with an increased density and intensity of TDO2-positive gray and white matter glial cells.	[249]
Ischemia	OBSCs	KMO inhibition-mediated attenuation of post-ischemic neuronal death.	[204]
		Sharp QUIN and 3-HK spikes in hippocampal slices under oxygen-glucose deprivation; prevention of neuronal death by pharmacological KMO inhibition (e.g., Ro 61-8048).	[251]

## Data Availability

No new data were created or analyzed in this study.

## Abbreviations

The following abbreviations are used in this manuscript:

1-MT	1-Metyltryptophan
3-HAA	3-Hydroxyanthranilic acid
3-HK	3-Hydroxykynurenine
8-OH-QUINA	8-Hydroxyquinaldic acid
Aβ	Amyloid-β
AA	Anthranilic acid
AADAT	Alpha-aminoadipate aminotransferase
ACMSD	Aminocarboxymuconate semialdehyde decarboxylase
AD	Alzheimer’s disease
AhR	Aryl hydrocarbon receptor
AMPA	α-amino-3-hydroxy-5-methyl-4-isoxazolepropionic acid receptor
AMSDH	Aminomuconate semialdehyde dehydrogenase
ATCC	American Type Culture Collection
BDNF	Brain-derived neurotrophic factor
CA	Cinnabaric acid
CD45	Protein tyrosine phosphatase receptor type C
CRISPR/Cas9	Clustered regularly interspaced short palindromic repeats/CRISPR associated protein 9
DNA	Deoxyribonucleic Acid
ERK	Extracellular signal-regulated kinase
GCDH	Glutaryl-CoA dehydrogenase
CNS	Central nervous system
GFAP	Glial fibrillary acid protein
GPR35	G protein-coupled receptor 35
HD	Huntington’s disease
hESC	Human embryonic stem cells
HIF	Hypoxia-inducible factor
Iba1	Ionized calcium-binding adapter molecule 1
IDO	Indoleamine 2,3-dioxygenase
IFN	Interferon
IL	Interleukin
iAstrocytes	iPSC-derived astrocytes
iMicroglia	iPSC-derived microglia
iNeurons	iPSC-derived neurons
iNOS	Inducible nitric oxide synthetase
iPSC	Induced pluripotent stem cells
KAT	Kynurenine aminotransferase
KMO	Kynurenine 3-monooxygenase
KP	Kynurenine pathway
KYN	L-Kynurenine
KYNA	Kynurenic acid
KYNU	Kynureninase
LPS	Lipopolysaccharide
MDD	Major depressive disorder
mRNA	Messenger ribonucleic acid
NG2	Neural/glial antigen 2
NGF	Nerve growth factor
NMDA	N-methyl-D-aspartate receptor
NO	Nitric oxide
nNOS	Neuronal nitric oxide synthetase
NAD^+^	Nicotinamide adenine dinucleotide
OBSC	Organotypic brain slice culture
PD	Parkinson’s disease
PDGFRβ	Platelet-derived growth factor receptor beta
PIC	Picolinic acid
PU.1	Transcription factor PU.1
QPRT	Quinolinate phosphoribosyltransferase
QUIN	Quinolinic acid
QUINA	Quinaldic acid
ROS	Reactive oxygen species
TDO	Tryptophan 2,3-dioxygenase
TNF	Tumor necrosis factor
TRP	L-Tryptophan
XANA	Xanthurenic acid
α7-nAChR	Alpha-7 nicotinic acetylcholine receptor
